# Combining Multiparametric MRI Radiomics Signature With the Vesical Imaging-Reporting and Data System (VI-RADS) Score to Preoperatively Differentiate Muscle Invasion of Bladder Cancer

**DOI:** 10.3389/fonc.2021.619893

**Published:** 2021-05-13

**Authors:** Zongtai Zheng, Feijia Xu, Zhuoran Gu, Yang Yan, Tianyuan Xu, Shenghua Liu, Xudong Yao

**Affiliations:** ^1^ Department of Urology, Shanghai Tenth People′s Hospital, Tongji University School of Medicine, Shanghai, China; ^2^ Department of Radiology, Shanghai Tenth People′s Hospital, Tongji University School of Medicine, Shanghai, China

**Keywords:** bladder cancer, muscle-invasive status, multiparametric magnetic resonance imaging, nomogram, radiomics, Vesical Imaging-Reporting and Data System

## Abstract

**Background:**

The treatment and prognosis for muscle-invasive bladder cancer (MIBC) and non-muscle-invasive bladder cancer (NMIBC) are different. We aimed to construct a nomogram based on the multiparametric MRI (mpMRI) radiomics signature and the Vesical Imaging-Reporting and Data System (VI-RADS) score for the preoperative differentiation of MIBC from NMIBC.

**Method:**

The retrospective study involved 185 pathologically confirmed bladder cancer (BCa) patients (training set: 129 patients, validation set: 56 patients) who received mpMRI before surgery between August 2014 to April 2020. A total of 2,436 radiomics features were quantitatively extracted from the largest lesion located on the axial T2WI and from dynamic contrast-enhancement images. The minimum redundancy maximum relevance (mRMR) algorithm was used for feature screening. The selected features were introduced to construct radiomics signatures using three classifiers, including least absolute shrinkage and selection operator (LASSO), support vector machines (SVM) and random forest (RF) in the training set. The differentiation performances of the three classifiers were evaluated using the area under the curve (AUC) and accuracy. Univariable and multivariable logistic regression were used to develop a nomogram based on the optimal radiomics signature and clinical characteristics. The performance of the radiomics signatures and the nomogram was assessed and validated in the validation set.

**Results:**

Compared to the RF and SVM classifiers, the LASSO classifier had the best capacity for muscle invasive status differentiation in both the training (accuracy: 90.7%, AUC: 0.934) and validation sets (accuracy: 87.5%, AUC: 0.906). Incorporating the radiomics signature and VI-RADS score, the nomogram demonstrated better discrimination and calibration both in the training set (accuracy: 93.0%, AUC: 0.970) and validation set (accuracy: 89.3%, AUC: 0.943). Decision curve analysis showed the clinical usefulness of the nomogram.

**Conclusions:**

The mpMRI radiomics signature may be useful for the preoperative differentiation of muscle-invasive status in BCa. The proposed nomogram integrating the radiomics signature with the VI-RADS score may further increase the differentiation power and improve clinical decision making.

## Introduction

Bladder cancer (BCa) remains one of the most commonly diagnosed cancer in urological diseases. According to the degree of tumor invasion, BCa is classified as either muscle-invasive bladder cancer (MIBC) or non-muscle-invasive bladder cancer (NMIBC). About 75% of newly diagnosed BCa patients have NMIBC while the remaining patients have MIBC (1).

Determining muscle invasion status is critical in treatment decision making. MIBC patients should receive radical cystectomy as the gold standard while NMIBC patients are treated to preserve the bladder (2). Therefore, accurately differentiating MIBC from NMIBC is critical for BCa patients. However, precisely diagnosing muscle invasiveness preoperatively is not an easy task.

Currently, the cystoscopic biopsy is commonly used for tumor diagnosis and clinical staging. However, this approach is invasive and expensive. In addition, it was reported that 20% to 80% of lesions were misdiagnosed due to variations in performing cystoscopic biopsy (3), and upstaging to MIBC occurred in 32% of cases that were diagnosed as NMIBC according to the initial cystoscopic biopsy (4). Magnetic resonance imaging (MRI) is usually used in the detection of BCa and is also increasingly used to preoperatively predict the muscle-invasive status (5, 6). Multiparametric MRI (mpMRI) can provide high spatial and contrast resolution images, regional anatomic structures and identification of the urinary bladder layers, which contribute to reducing staging errors (5, 6). Several sequences including conventional T1- (T1WI) and T2-weighted imaging (T2WI) and more advanced sequence such as dynamic contrast-enhancement (DCE) and diffusion-weighted imaging (DWI) has demonstrated reliable results for diagnosing muscle invasiveness of BCa (7–10). However, this approach is expertise-dependent, and its diagnostic performance is not sufficiently accurate (9, 11). In addition, there currently insufficient data on the use of advanced MRI techniques to allow for a recommendation to be made in the guidelines (12).

The Vesical Imaging-Reporting and Data System (VI-RADS), based on mpMRI, was released in 2018 and is regarded as an imaging protocol and reporting criterion for bladder MRI which provides a more meticulous distinction between clinical stages that were previously difficult to differentiate by conventional MRI interpretation (3). The integration of T2WI, DWI, and DCE is the cornerstone for standardizing the VI-RADS reporting system. VI-RADS provides five-point scores that predict the possibility of muscle invasiveness by BCa. The reported accuracy of VI-RADS in predicting MIBC has exceeded 85% in recent validation studies (13–17) with a great inter-reader agreement and reviewer acceptance (17, 18), so VI-RADS has obtained novel interest and acceptance and has been adopted by many radiologists and institutions in clinical routine. Recent studies reported that VI-RADS also had the potential to differentiate BCa patients with extravesical extension (19) and select high risk NMIBC patients who are a candidate for repeated transurethral Resection (16). Despite its promising prospects, VI-RADS still relies on experienced radiologists, which could inevitably result in human error.

An additional objective method is radiomics, which converts medical images into quantitative mineable data that are subsequently analyzed with artificial intelligence, applying the useful features to guide clinical decision making. It has recently drawn great attention for the preoperative prediction of tumor staging, lymph node metastasis, prognosis, therapeutic response and muscle-invasive status (20–24). Moreover, DCE modality has conventionally been considered useful for pathological staging and histological grading in bladder cancer (9), but the radiomics signature of DCE has never been analyzed in previous studies to the best of our knowledge.

Therefore, in our study, we aimed to 1) develop and validate radiomics signatures from T2WI and DCE modalities to identify muscle invasion in BCa, and 2) construct a nomogram integrating the VI-RADS score and radiomics signature to improve differentiation power.

## Materials and Methods

### Ethics

The studies involving human participants were reviewed and approved by the Ethics Committee of Shanghai Tenth People’s Hospital.

### Patients

We retrospectively collected 185 patients with surgical resection of a pathologically confirmed BCa from August 2014 to April 2020 at our institution. Due to its retrospective nature, the informed consent of patients was waived. The inclusion and exclusion criteria of our study were presented in **Supplementary Figure 1**. We randomly allocated 7/10 of eligible patients to the training set and the remaining to the validation set in a 7:3 ratio.

### Image Acquisition

All examinations were performed using a 3-T MRI scanner (Magnetom Verio: Siemens, Erlangen, Germany), equipped with an 8-channel phased-array coil. Axial T1WI (TR/TE, 600/11), turbo-spin-echo nonfat-suppressed T2WI with a slice thickness of 4 to 6 mm in axial and coronal planes and turbo-spin-echo fat-suppressed T2WI in the sagittal plane with a slice thickness of 6 mm was performed. Axial DCE fat-suppressed T1WI with a slice thickness of 3mm were performed after injection of Gadopentetate administered at a dose of 0.1 mmol/kg at a rate of 1.5 to 2 ml/s. Five to six sets of CE images including three orthogonal planes were acquired 20 to 131 s after the injection of contrast agents. Pre-contrast imaging was also needed. DWI was performed with breathing-free spin-echo echo planar imaging sequence in axial including high b value (800–1,000 s/mm^2^) to display BCa with high contrast to surrounding tissues. Due to the inconsistency of the b value, the DWI images were not included in the study.

### VI-RADS Score Evaluation

**Figure 1:**
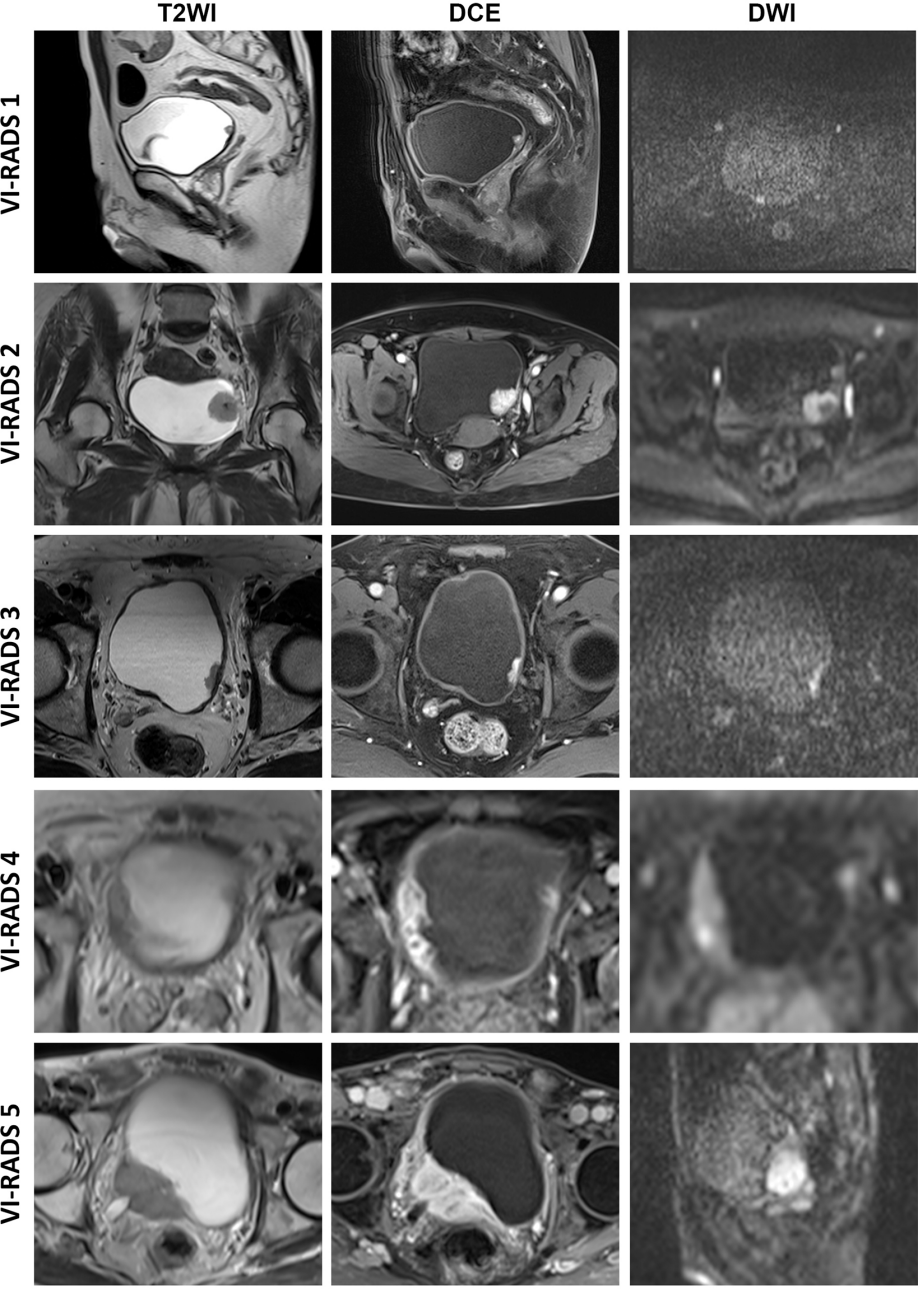
Typical images of VI-RADS scores. VI-RADS, Vesical Imaging-Reporting and Data System; DCE, dynamic contrast-enhancement; DWI, diffusion-weighted imaging.

Two experienced radiologists (F Xu and T Xu), familiar with the VI-RADS algorithm (3) and blinded to the patients’ clinical information, independently evaluated the MRI images based on the 5-point VI-RADS scoring system (**Figure 1**). Tumor size and the number of tumors were recorded based on the schematic map. For patients with multiple lesions, the lesion with the maximal diameter in the bladder lumen was selected and measured, and the VI-RADS score was considered the highest one. Discordance between the VI-RADS scores of the two radiologists was carefully corrected by consensus.

### Region of Interest (ROI) Segmentation

**Figure 2:**
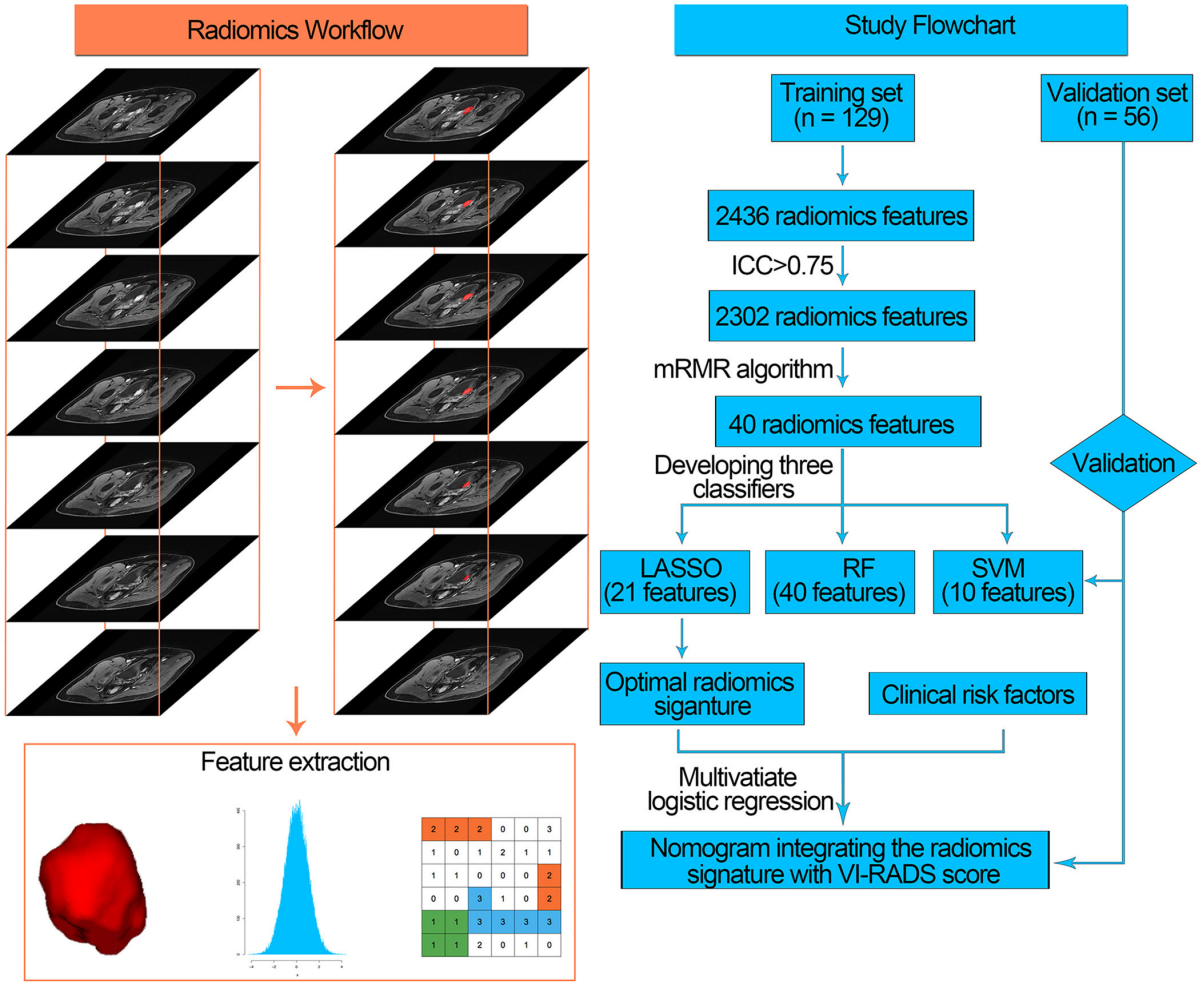
Radiomics workflow (left) and study flowchart (right) of our study. mRMR, minimum redundancy maximum relevance, ICC, intra- and interclass correlation coefficient; LASSO, least absolute shrinkage and selection operator; RF, random forest; SVM, support vector machine; VI-RADS, Vesical Imaging-Reporting and Data System.

One radiologist (F Xu) with bladder MRI reading experience of over 5 years manually drew tumor ROIs along the edges of the lesion on each slice for the entire tumor with the maximal diameter in each patient’s bladder lumen (**Figure 2**). Then, all ROIs were merged for the whole tumor volume ROI. Volumes of interest (VOIs) were then manually segmented on T2WI images and during the fifth phase of DCE images (60 s after injection of the contrast agent) via a free open-source software package (ITK-SNAP, version 3.6.0; http://itk-snap.org). After 30 days, the VOIs of 40 randomly selected patients were repeatedly segmented by the same radiologist and another radiologist (T Xu) for intra- and inter-observer repeatability tests. The intra- and interclass correlation coefficients (ICCs) were used to evaluate the intra- and inter-observer agreement on feature extraction.

### Feature Extraction

After segmenting the ROI of the tumor, radiomics features were extracted applying the PyRadiomics platform which can extract standardized radiomics features from medical images (http://www.radiomics.io/pyradiomics.html) (25). In this study, we identified four classes of imaging features, including shape and size-based features, image intensity (first-order features), textural features and wavelet features. In total, 2,436 radiomics features were extracted from axial T2WI and DCE images using the PyRadiomics platform. Each radiomics feature was then normalized into its Z-score.

### Feature Screening and Radiomics Signature Construction

Feature screening not only serves as a dimension-reduction approach but also selects features that could provide deeper insight into the differentiation task. ICC was calculated for the extracted radiomics features, and features with ICC>0.75 were selected for further analysis.

Then, we used the minimum redundancy maximum relevance (mRMR) algorithm to rank features with mutual information (MI). The mRMR algorithm is a supervised feature selection model which initially calculates the MI between features and a target variable. It ranks the features via maximizing MI with respect to the target variable and then minimizes the average MI for features with higher rankings. In this way, the top 40 features were selected.

Subsequently, these 40 top-ranking radiomics features were introduced into a classifier to construct a radiomics signature for muscle-invasive status differentiation. In this study, we developed three classifiers for muscle-invasive status differentiation, including the least absolute shrinkage and selection operator (LASSO), random forest (RF), and support vector machine (SVM) algorithms.

LASSO adds the penalty for non-zero coefficients to the sum of the absolute value (L1 penalty). The features that minimally influence the target variable are removed, and the features with non-zero coefficients were selected. The radiomics score of the LASSO classifier was calculated by summing the selected radiomics features weighted by their coefficients.

A nonlinear SVM-based recursive feature elimination (SVM-RFE) algorithm was performed to determine the optimal subset of features for SVM classifier construction. The goal of the SVM-RFE algorithm was to rank and select features. The selection process included backward elimination in each iteration, wherein features that had the least impact on improving the differentiation power of the classifier were omitted. In addition, the SVM-RFE algorithm was used to investigate the optimal number of radiomics features to develop a SVM classifier with the highest accuracy.

Random Forest-Feature Selection (RFS-FS) was used to rank feature importance according to the Mean Decrease in the Gini index and to determine the optimal number of features to develop an RF classifier with the lowest differentiation error.

The three classifiers were all trained using 10-fold cross-validation on the training set to determine the optimal parameter configuration for each classifier and were then tested on the validation set. The differentiation performance of each classifier was compared applying the receiver operating characteristic (ROC) curve and calculated by the area under the curve (AUC). Accuracy, sensitivity, specificity, negative-predictive value (NPV), and positive-predictive value (PPV) both in the training and validation sets were calculated based on the Youden index (26). The radiomics classifier with the highest AUC and accuracy was regarded as the optimal radiomics signature.

### Combination Model and Nomogram Development

The clinical characteristics, including age, sex, MRI-determined number of tumors, MRI-determined tumor size and VI-RADS score, were prepared for building a combination model on the training set. These factors, together with the radiomics score generated from the optimal radiomics signature, were tested using univariate analysis. Significant factors in the univariate analysis were put into a step-wise multivariate logistic regression analysis to develop a combination model applying the likelihood ratio test. The coefficients of factors selected by the multivariate logistic regression were applied to develop a nomogram. Variance inflation factors (VIF) were calculated to diagnose the collinearity of the multivariate logistic regression.

Decision curve analysis (DCA) was utilized to investigate the clinical utility of the nomogram for decision making. Sensitivity, specificity, accuracy, AUC, and calibration curves were employed to evaluate the performance of the nomogram. Harrell’s concordance index (C-index) and Hosmer-Lemeshow test were performed to quantitatively measure the degree of fit of the nomogram. In addition, we used integrated discrimination improvement (IDI) and net reclassification improvement (NRI) to investigate the incremental diagnostic utility of the nomogram compared with VI-RADS scores (27).

## Statistical Analysis

Statistical analysis was conducted with R statistical software (version 3.6.1 R, https://www.r-project.org/). R packages used for statistical analysis were listed in the **Supplementary Table S1**. The clinical factors between the training and validation sets were compared applying the Student’s t-test, the Chi-square test, or the Mann-Whitney U test, as appropriate. Differences in the radiomics score among multiple groups were evaluated using one−way ANOVA followed by Dunnett’s post−hoc test. A forward stepwise selection was used with Akaike’s information criterion (AIC) as the stopping rule. In this study, muscle invasion was regarded as positive. All differentiation classifiers were developed on the training set and validated on the validation set. All tests were 2-tailed, and P values<0.05 were regarded as statistically significant.

## Results

### Patient Population

One hundred and eighty-five patients (123 NMIBC patients and 62 MIBC patients) were randomly separated into a training set (129 patients) and a validation set (56 patients). The characteristics of patients in the two data sets were listed in **Table 1**. There were no significant differences in clinical factors including patients’ sex, age, tumor size, tumor number, VI-RADS scoring and MIBC (≥pT2) prevalence between the training and validation sets.

**Table 1:** Baseline patient characteristics.

Characteristic	Number of Patients (%)		*P* value
	Training Set (n = 129)	Validation Set (n = 56)	
Sex			
Men	109 (84.5)	44 (78.5)	0.398^a^
Women	20 (15.5)	12 (21.4)	
Age (years)			
<65	48 (37.2)	20 (35.7)	0.870^a^
≥65	81 (62.8)	36 (64.3)	
MRI-determined tumor size (cm)			
<3	82 (63.6)	37 (66.1)	0.868^a^
≥3	47 (36.4)	19 (33.9)	
MRI-determined number of tumors			
Single	89 (69.0)	38 (67.9)	0.865^a^
Multiple	40 (31.0)	18 (32.1)	
VI-RADS score			
1	18 (14.0)	7 (12.3)	0.499^b^
2	36 (27.9)	12 (21.1)	
3	33 (25.6)	19 (33.3)	
4	20 (15.5)	6 (10.5)	
5	22 (17.1)	12 (21.1)	
Pathologic tumor (pT) stage			
<pT2	85 (65.9)	38 (66.7)	0.866^a^
≥pT2	44 (34.1)	18 (31.6)	

MRI, magnetic resonance imaging; VI-RADS, Vesical Imaging-Reporting and Data System.

a Statistical analysis performed using chi-square test.

b Statistical analysis performed using Mann-Whitney U test.

### Radiomics Signature Construction

Based on the standard of ICC>0.75 in the intra- and inter-observer tests, 1,136 features from DCE images and 1,166 features from T2WI images were highly robust and selected for subsequent analysis. The mRMR algorithm was performed to rank features according to their relevance-redundancy index and filter out the redundant and irrelevant features, from which the top 40 features were retained. Subsequently, the RF, SVM, and LASSO classifiers were trained on the training set using the top 40 features. The differentiation abilities of radiomics classifiers were tested on the validation set.

**Figure 3:**
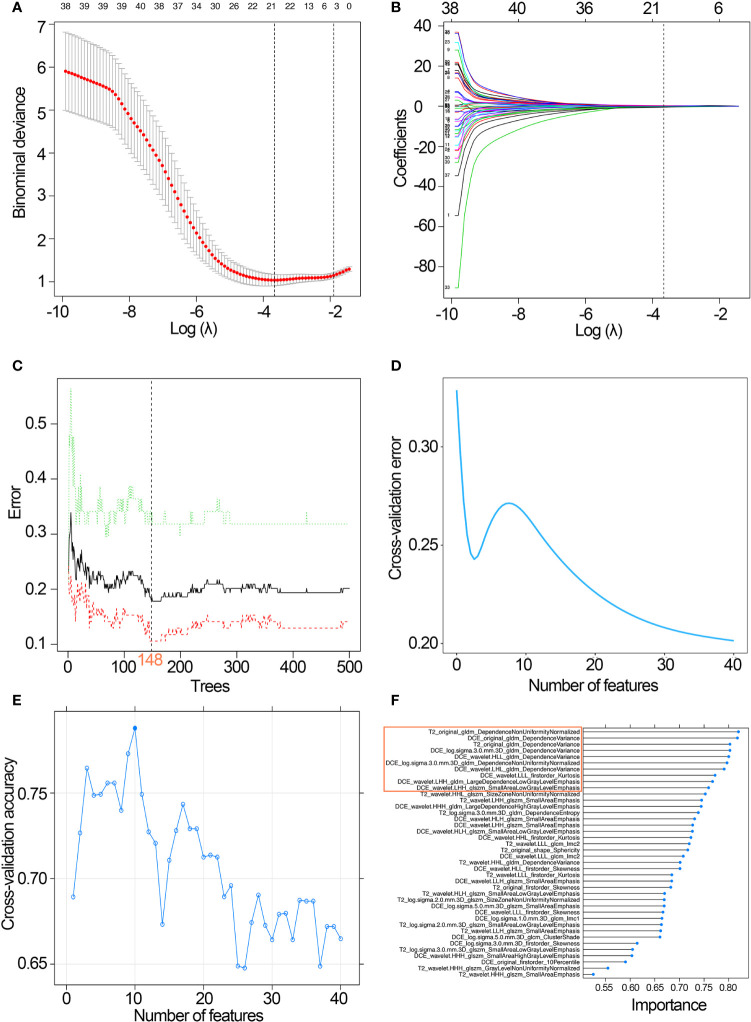
Development of the three classifiers. **(A)** Selection of the tuning parameter λ in the LASSO classifier *via* 10-fold cross-validation based on minimum criteria. **(B)** LASSO coefficient profiles of the 21 radiomics features. A vertical line was drawn at the selected value, where the optimal λ resulted in eight non-zero coefficients. **(C)** Selection of the optimal number of growing trees (ntree=148) with the lowest discriminative error in the RF classifier. **(D)** Feature selection process using RFS-FS and 10-fold cross-validation in the training set: 40 features with the lowest discriminative error were selected for predictive classifier development. **(E)** Feature selection process using SVM-RFE and 10-fold cross-validation in the training set: 10 features with the highest discriminative accuracy were selected for SVM classifier development; **(F)** SVM-RFE is used to rank features according to the feature importance, and the top 10 features were selected for SVM classifier development. LASSO, least absolute shrinkage and selection operator; RF, random forest; SVM, support vector machine; RFS-FS, RF-feature selection; SVM-RFE, SVM-based recursive feature elimination.

The LASSO classifier was performed to select the optimized subset of features and calculate the radiomics score for each patient. Twenty-one features with non-zero coefficients were screened based on minimum criteria (**Figures 3A, B** **)**. The coefficients and the calculation formula were presented in **Supplementary Figure 2**.

With 40 features chosen by the RFS-FS algorithm, an RF classifier with the lowest cross-validation error was developed via 148 growing trees (**Figures 3C, D** **)**.

The top 10 features selected by the SVM-RFE algorithm were then used to build an SVM classifier with the highest accuracy for evaluating the muscular invasiveness of BCa (**Figures 3E, F** **)**.

### Performance of the Radiomics Signatures for Muscle-Invasive Status Differentiation

**Figure 4:**
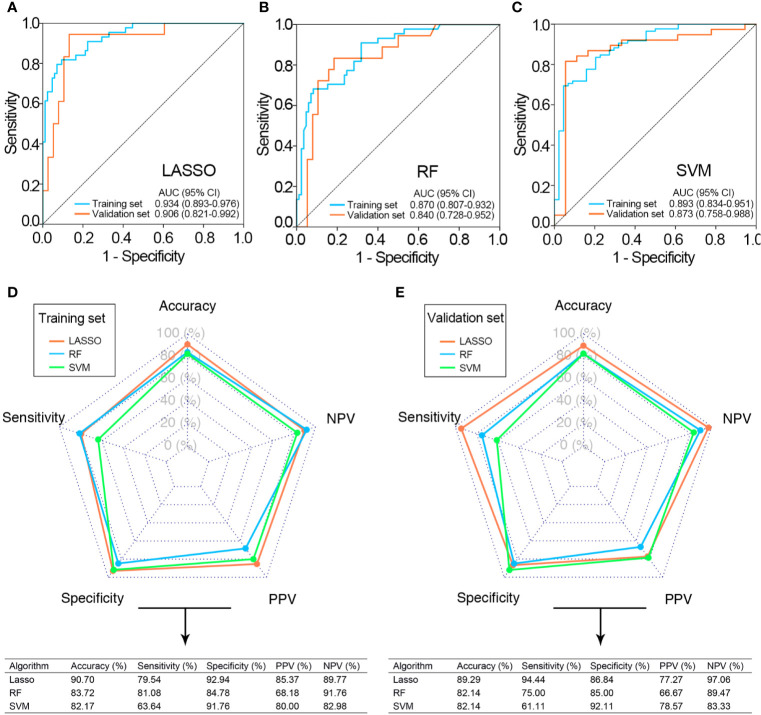
Performance of three classifiers for the preoperative differentiation of muscle-invasive status. ROC curves of LASSO **(A)**, RF **(B)**, and SVM classifiers **(C)** in the training and validation sets. The predictive performance of the three classifiers in the training **(D)** and validation sets **(E)**. CI, confidence interval; ROC, receiver operating curve; AUC, area under the ROC curve; LASSO, least absolute shrinkage and selection operator; RF, random forest; SVM, support vector machine; NPV, negative predictive value; PPV, positive predict value.

The performance of three radiomics signatures (LASSO, SVM, and RF classifiers) for muscle-invasive status differentiation was showed in **Figure 4**. The SVM and RF classifiers led to relatively consistent performance, while the LASSO classifier had more capacity in muscle-invasive status differentiation both in the training and validation sets. In this way, the optimal radiomics signature generated by the LASSO classifier was selected for further analysis.

Among the 21 features selected for the LASSO classifier, 10 of them were from T2WI and 11 of them were from DCE. Furthermore, these features were not highly correlated with each other (Pearson correlation coefficients ranging from 0.005 to 0.325; **Supplementary Figure 3**).

The optimal cutoff value for radiomics score was −0.093 determined in the training set. According to the optimal cutoff value, the accuracy and AUC of the radiomics signature in muscle-invasive status differentiation were 90.7% and 0.934 (95% confidence interval (CI): 0.893, 0.976, P value<0.01) in the training set and 87.5% and 0.906 (95% CI: 0.821, 0.992, *P* value<0.01) in the validation set, respectively (**Figures 4A, D, E** **)**.

**Figure 5:**
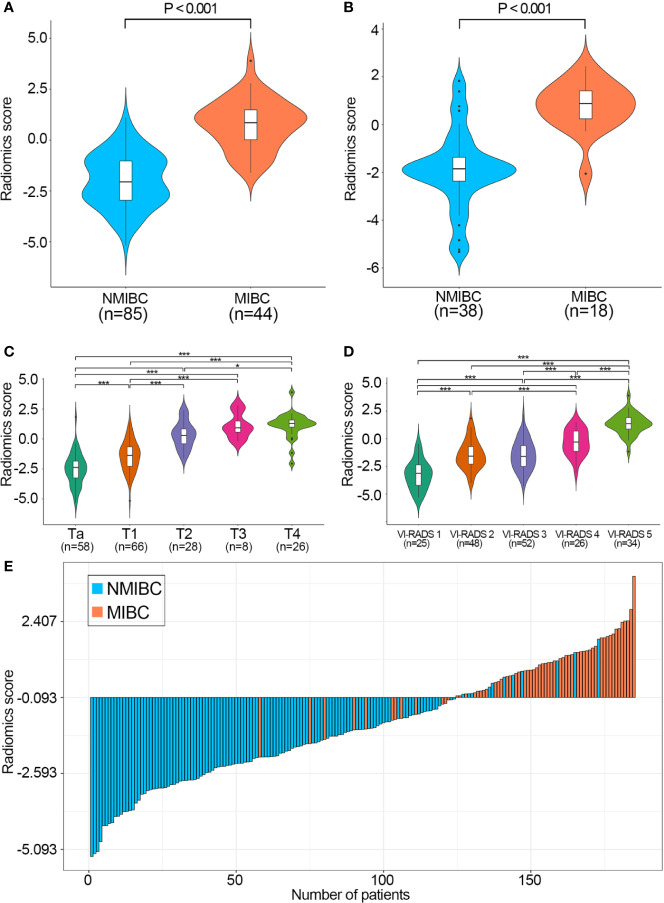
Performance of the radiomics score calculated by the LASSO classifier. Boxplots of the radiomics score in the training **(A)** and validation sets **(B)** grouped by the muscle-invasive status. Boxplots of the radiomics score in the combined training and validation set grouped by T stage **(C)** and VI-RADS score **(D)**, respectively. P values were calculated using one−way ANOVA followed by Dunnett’s post−hoc test. **(E)** Waterfall plot of the distribution of radiomics scores and pathologic tumor stages of individual patients in the combined training and validation sets. LASSO, least absolute shrinkage and selection operator; VI-RADS, Vesical Imaging-Reporting and Data System; NMIBC, non-muscle-invasive bladder cancer; MIBC, muscle-invasive bladder cancer. *P < 0.05, **P < 0.001.

NMIBC patients had significantly higher radiomics scores than MIBC patients in both data sets (**Figures 5A, B** **)**. In addition, patients with higher T stages or VI-RADS scores had significantly higher radiomics scores in the combined training and validation set (**Figures 5C, D** **)**.

The waterfall plot showed that patients with high radiomics scores had a strong tendency for muscle invasion in the combined training and validation sets, which indicated that the muscle-invasive status of BCa patients could be correctly predicted based on the cutoff value of the radiomics signature (**Figure 5E**).

We further evaluated the performance of the radiomics signature in 52 BCa patients with VI-RADS scores of 3. The radiomics signature exhibited a relatively favorable differentiation with an accuracy of 86.5% and an AUC of 0.833 (95% CI: 0.650–1.000, *P* value<0.01) (**Supplementary Figure 4A**). The DCA plot indicated that the radiomics signature had the highest clinical net benefit with wider threshold probabilities compared with other clinical factors in this subgroup (**Supplementary Figure 4B**).

### Development and Performance of the Nomogram

The important clinical factors and the radiomics score calculated by radiomics signature were investigated applying univariate and multivariate regression (**Table 2**). Three factors, including MRI-determined tumor size, VI-RADS and radiomics scores were significantly associated with BCa muscle-invasive status (*P*<0.001) in the univariate regression. After multivariate analysis, the radiomics score and VI-RADS remained strong independent predictors for muscle-invasive status differentiation with the lowest AIC value (AIC=58.86). Regarding the collinearity diagnosis, the VIF of candidate predictors ranged from 1.660 to 2.754, indicating that there was no collinearity. The risk score was calculated based on the formula as follows: (1.385 × radiomics score) + (1.796 × VI-RADS score) − 5.648.

**Table 2:** Univariate and multivariable regression analyses of the radiomics score and clinical factors in the training cohort.

Variables	Univariate analysis			Multivariate analysis		
	β	OR (95% CI)	P value	β	OR (95% CI)	P value
Sex: Men vs women	0.514	1.671 (0.565–4.958)	0.354	–	–	–
Age (continuous), year	−0.015	0.985 (0.956–1.016)	0.351	–	–	–
MRI-determined number of tumors, number	−0.095	0.910 (0.782–1.058)	0.220	–	–	–
MRI-determined tumor size (continuous), cm	0.678	1.970 (1.479–2.625)	<0.001	–	–	–
VI-RADS score	2.295	9.920 (4.566–21.553)	<0.001	1.796	6.025 (2.417–15.022)	<0.001
Radiomics score	1.756	5.788 (3.115–10.754)	<0.001	1.385	3.996 (1.824–8.756)	0.001

OR, odds ratio; MRI, magnetic resonance imaging; VI-RADS, Vesical Imaging-Reporting and Data System.

**Figure 6:**
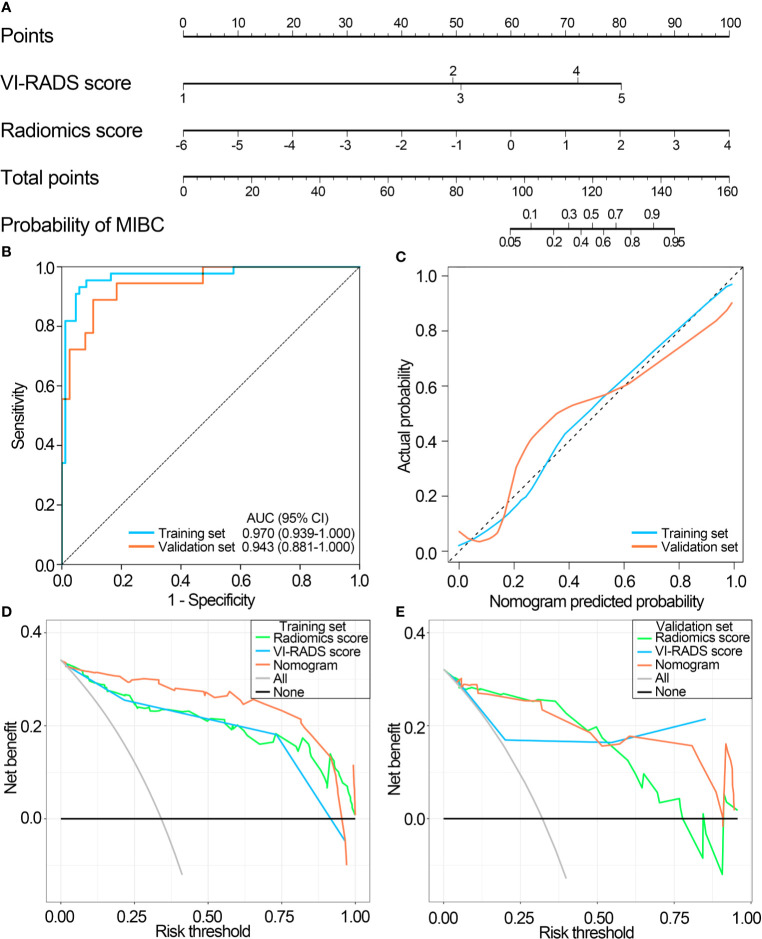
Performance of the nomogram integrating the radiomics score with the VI-RADS score. **(A)** The nomogram integrating the radiomics score with the VI-RADS score was constructed to predict muscular invasiveness in patients with bladder cancer. **(B)** ROC curves of the nomogram in the training and validation sets. **(C)** Calibration curve of the nomogram in the training and validation sets. **(D)** DCA for radiomic signature, VI-RADS score and nomogram in the training set. **(E)** DCA for radiomic signature, VI-RADS score and nomogram in the validation set. VI-RADS, Vesical Imaging-Reporting and Data System; ROC, receiver operating curve; AUC, area under the ROC curve; DCA, decision curve analysis.

Then, a nomogram was generated by incorporating the radiomics score and VI-RADS score for muscle-invasive status differentiation (**Figure 6A**). The nomogram further improved the differentiation power with the AUC of 0.970 (95% CI, 0.939–1.000, *P* value < 0.01) in the training set and 0.943 (95% CI, 0.881–1.000, *P* value < 0.01) in the validation set (**Figure 6B**). The calibration plots suggested marked concordance between prediction and observation both in the training set and validation set (**Figure 6C**). Harrell’s C-indices of the nomogram were 0.966 (95% CI, 0.945–0.987) and 0.906 (95% CI, 0.874–0.938) in the training and the validation sets, respectively. The Hosmer-Lemeshow test yielded nonsignificant *P* values of 0.537 and 0.929 in the training and the validation sets, respectively, indicating good calibration power.

The DCA plots showed that the radiomics score and the VI-RADS score had a relatively consistent clinical net benefit, and the nomogram combining radiomics score and VI-RADS score had the highest clinical net benefit both in the training set and validation set (**Figures 6D, E** **)**. Furthermore, compared with the VI-RADS score, the nomogram also significantly improved diagnostic accuracy for muscle-invasive status differentiation (overall category-based NRI, 0.325; NRI indices for events and nonevents, 6.5% and 26.90%, respectively; IDI, 0.118, *P*<0.001), and similar results were also observed in the training set and the validation set, respectively, which were showed in **Table 3**.

**Table 3:** The NRI and IDI indices.

	Combined training and validation set		Training set		Validation set	
	Values	P value	Values	P value	Values	P value
NRI	0.325	<0.001	0.280	<0.001	0.450	0.001
Events NRI	6.45%		6.82%		5.56%	
Nonevents NRI	26.02%		21.18%		39.47%	
IDI	0.118	<0.001	0.115	<0.001	0.128	0.001

NRI, net reclassification improvement; IDI, integrated discrimination improvement.

## Discussion

In this study, we sought to evaluate the ability of mpMRI radiomics features extracted from DCE and T2WI to discriminate MIBC from NMIBC. For each mpMRI modality, we extracted most of the radiomics features mentioned in the current literature. After evaluating reproducibility by ICC and eliminating redundancies by mRMR, we used the remaining features to develop radiomics signatures. Three mpMRI radiomics signatures (LASSO, RF, and SVM) were constructed and validated for the preoperative differentiation of MIBC from NMIBC.

The results showed that the optimal radiomics signature, generated by the LASSO classifier, achieved favorable differentiation performances in the training and validation sets for the prediction task. As a VI-RADS score of 3 is an equivocal category for muscle-invasive status differentiation, we further investigated the performance of the radiomics signature in BCa patients with VI-RADS scores of 3. The results suggested that the radiomics signature still had favorable discriminatory power in this subgroup. In addition, a nomogram integrating the optimal radiomics signature with the VI-RADS score could further improve the discriminatory power and obtain good calibration and favorable clinical net benefit, suggesting a promising and noninvasive clinical tool for muscle-invasive status prediction.

Muscular invasiveness in patients with BCa indicates a negative prognosis, and the muscle-invasive status is critical for treatment decision-making in patients with BCa (2). Currently, a cystoscopic biopsy of suspicious bladder lesions is recommended for preoperative T staging and muscle-invasive status identification in BCa (28), but diagnostic sensitivity and accuracy are frequently unsatisfactory. The incompleteness of transurethral resection, absence of detrusor muscle, delay in the interval from transurethral resection to radical cystectomy, and low sensitivity of preoperative staging approaches all can lead to misdiagnosis (28–30). This phenomenon may result in poor outcomes due to the high rate of progression and metastasis in MIBC (2). Hence, it is important to improve the accuracy of tumor staging, which may optimize disease treatment and improve outcomes for patients.

The mpMRI is regarded as an important tool for assessing the depth of invasion in BCa. However, the evaluation of mpMRI depends mostly on several tumor characteristics, such as the tumor size, tumor density, regularity of tumor margins, the pattern of enhancement and anatomic association with the surrounding tissues, which is expertise-dependent and subjective (31).

Radiomics can recognize subtle differences in intensity distribution which cannot be easily discovered by human eyes and can comprehensively characterize the tumor phenotype in medical images based on high throughput quantitative image features extracted from MRI or CT images. In the optimal radiomics signature (LASSO classifier), 12 of 21 radiomics features were obtained from wavelet filtered features which implied that the wavelet transform filter was a multiscale analytical method that could be used to investigate tumor morphology and pathophysiology on multiple scales. Wavelet transform filter generates eight decompositions per level (all possible combinations of applying either a high or a low pass filter) in each of the three dimensions (25). Wavelet filtered features were high-dimensional radiomics features that could not be easily deciphered by humans. Compared with visual inspection by radiologists or low-level radiomics features, the wavelet filtered features have a more underlying relationship with heterogeneity and tumor biology in various cancers, including renal cell carcinoma, prostate carcinoma, and intrahepatic cholangiocarcinoma (32–34).

We sought to construct a more effective radiomics signature and improve upon the previous radiomics methodology in multiple ways. First, we extracted four classes of imaging features, including shape and size-based features, image intensity (first-order features), textural features and wavelet features, which can comprehensively describe the local, regional, and global tissue heterogeneity of BCa. Second, DCE and T2WI radiomics features were extracted from a 3-dimensional region rather than a two-dimensional region. In this way, the intrinsic features of the lesions could be effectively described. Third, the optimal radiomics signature was developed based on the mpMRI radiomics features from DCE and T2WI. Compared with CT, mpMRI can provide different forms of soft-tissue contrast, as well as functional parameters, and provides a comprehensive evaluation of BCa. Specifically, T2WI permits the evaluation of the size and morphology of lesions. DCE reflects the microvessel permeability and issue vascularity of lesions, and the slight submucosal linear enhancement is regarded as a useful characteristic for the nonmuscle invasiveness condition of BCa (8). Our study used the DCE for radiomics signature development and muscle-invasive status identification in BCa and showed that the radiomics signature based on the T2WI and DCE radiomics features had a better discriminatory power compared with previous research based on MRI (23, 35, 36). Fourth, considering that clinical characteristics, such as sex, age, tumor size, and tumor number are commonly applied for the preoperative diagnosis of BCa patients, and given that the mpMRI-based VI-RADS score has been reported to be closely related to muscle-invasive status (37), we evaluated the diagnostic value of incorporating these clinical characteristics and the radiomics signature for the preoperative discrimination of muscle-invasive status. Our study demonstrated that the proposed nomogram integrating the radiomics signature with the mpMRI-based VI-RADS score further improved differentiation of muscle invasion in BCa. Calibration plots and DCA plots demonstrated good calibration and favorable clinical net benefit of the nomogram. The performance of this nomogram was superior to previous nomograms based on MRI images and clinical factors (23, 36). Finally, the software we used is publicly available and the PyRadiomics platform is open source for the radiomics procedure so that other institutions can apply and validate the proposed nomogram.

Some limitations in our study should be mentioned. First, due to the retrospective nature of our study, potential selection biases may have occurred. Prospective clinical trials are warranted. Second, the validation set drawn from the same institution prevented our study from evaluating the generalizability of the proposed nomogram to other institutions. Further external validation from different institutions is needed to determine the performance of the nomogram. Third, due to the lack of prognostic information, the correlation between the proposed nomogram and outcomes of BCa patients could not be determined.

In conclusion, our study developed a reliable mpMRI-based radiomics signature for preoperative discrimination of the muscle-invasive status in BCa. The proposed nomogram integrating the radiomics signature and the VI-RADS score further improved the discriminatory power and may provide added value for clinical decision making in BCa. Prospective clinical trials and multicenter studies are warranted to validate our results.

## Data Availability Statement

The processed data required to reproduce these findings cannot be shared at this time as the data also form part of an ongoing study. Requests to access the datasets should be directed to SL, drfelixliu@163.com.

## Author Contributions

XY, ZZ, and SL conceived and designed the study. XY and SL acquired the funding. ZZ, SL, FX, ZG, and TX collected and collated the data. ZZ, ZG, and FX were involved in the analysis and interpretation of data. SL, TX, and YY verified the underlying data. ZZ wrote the manuscript. XY, SL, and YY critically reviewed and revised the manuscript. ZZ, FX, and ZG designed the tables and figures. All authors read and approved the manuscript and agreed to be accountable for all aspects of the research in ensuring that the accuracy or integrity of any part of the work were appropriately investigated and resolved. All authors contributed to the article and approved the submitted version.

## Funding

This study was funded by the Outstanding Talent of Shanghai Tenth People's Hospital (20215YPDRC048), Shanghai Science Committee Foundation (grant number 19411967700) and Shanghai Youth Science and Technology Talents Sailing Program (20YF1437200).

## Conflict of Interest

The authors declare that the research was conducted in the absence of any commercial or financial relationships that could be construed as a potential conflict of interest.

## Supplementary Material

Supplementary Figure 1 Inclusion and exclusion criteria and recruitment pathways for patients in this study. MRI, magnetic resonance imaging.

Supplementary Figure 2 The coefficients and formula of the radiomics signature. **(A)** Histogram showing the coefficients of 21 selected features in the radiomics signature. **(B)** The formula for calculating the radiomics score of each patient.

Supplementary Figure 3 Pairwise Spearman rank correlation among 21 selected features in the training set.

Supplementary Figure 4 Performance of the radiomics score in 52 bladder cancer patients with VI-RADS score of 3. **(A)** ROC curves of the radiomics score in bladder cancer patients with VI-RADS score of 3. **(B)** DCA for radiomics score and other clinical factors in bladder cancer patients with VI-RADS score of 3. VI-RADS, Vesical Imaging-Reporting and Data System; ROC, receiver operating curve; AUC, area under the ROC curve; DCA, decision curve analysis.
