# Supplementary figures and images for: Combining Multiparametric MRI Radiomics Signature With the Vesical Imaging-Reporting and Data System (VI-RADS) Score to Preoperatively Differentiate Muscle Invasion of Bladder Cancer
Source: Front Oncol. 2021 May 13;11:619893. doi: 10.3389/fonc.2021.619893 (PMC8155615; doi:10.3389/fonc.2021.619893)

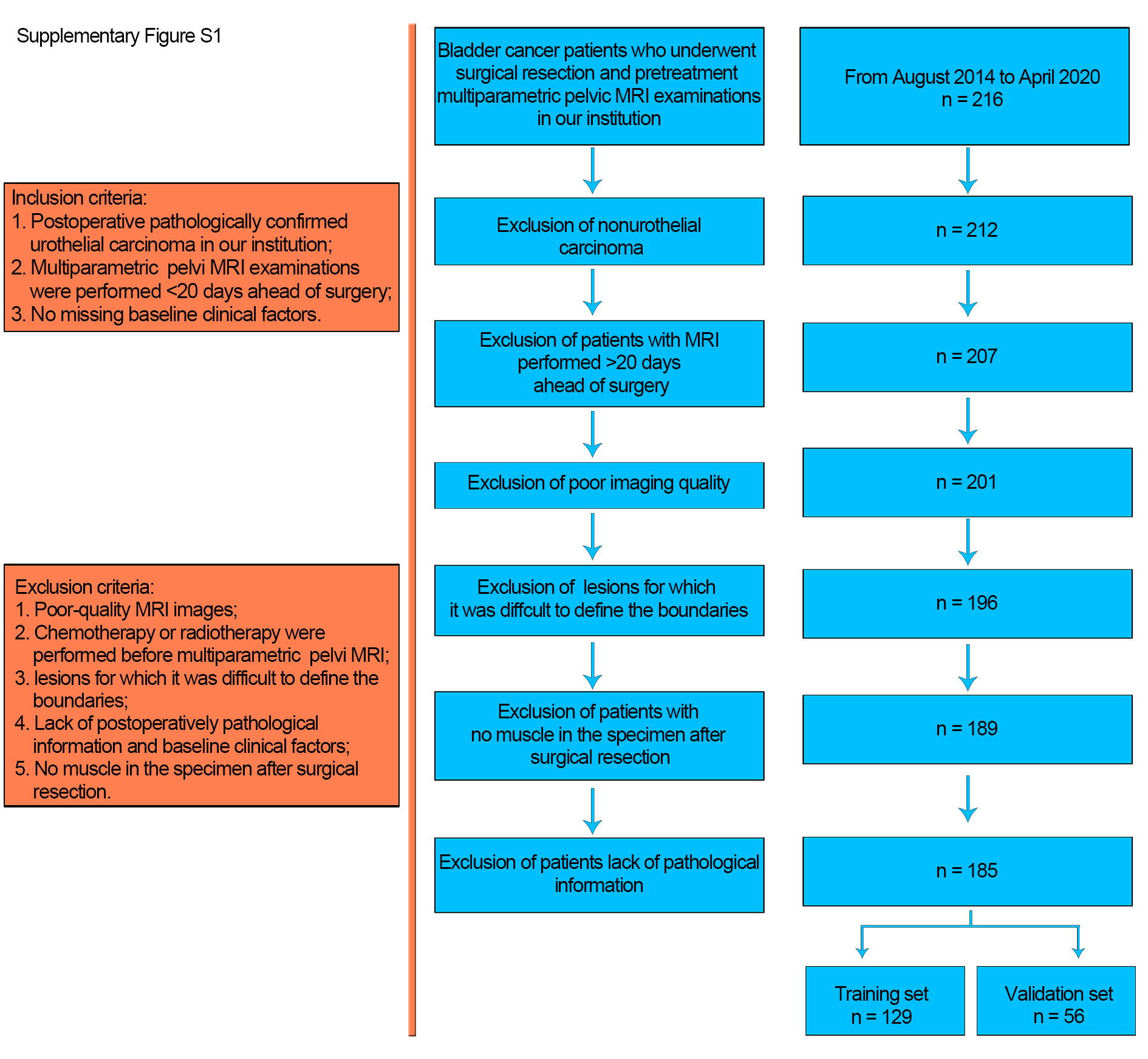

Supplement: Supplementary Figure 1 — Inclusion and exclusion criteria and recruitment pathways for patients in this study. MRI, magnetic resonance imaging. [file Image_1.jpeg]

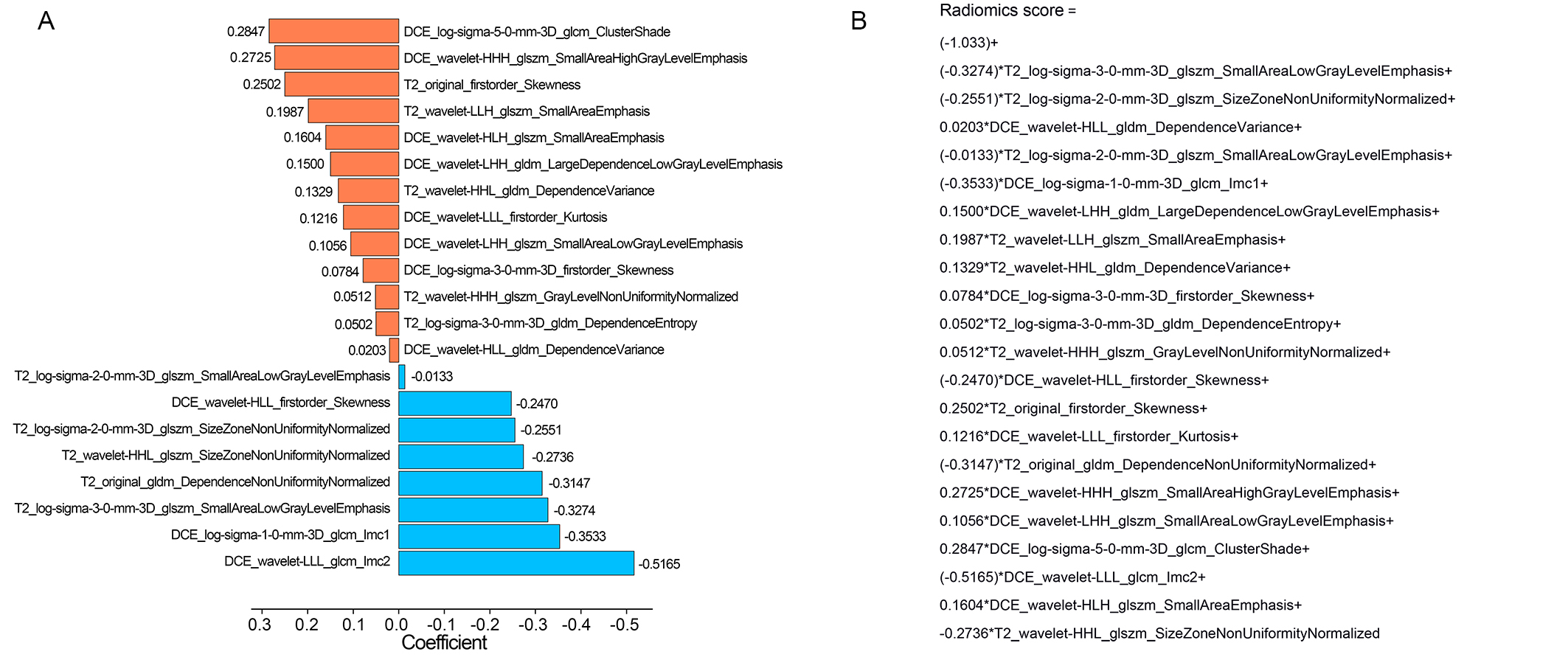

Supplement: Supplementary Figure 2 — The coefficients and formula of the radiomics signature. (A) Histogram showing the coefficients of 21 selected features in the radiomics signature. (B) The formula for calculating the radiomics score of each patient. [file Image_2.jpeg]
