# Supplementary material for: Combining Multiparametric MRI Radiomics Signature With the Vesical Imaging-Reporting and Data System (VI-RADS) Score to Preoperatively Differentiate Muscle Invasion of Bladder Cancer
Source: Front Oncol. 2021 May 13;11:619893. doi: 10.3389/fonc.2021.619893 (PMC8155615; doi:10.3389/fonc.2021.619893)
Supplement: Supplementary file 5 [file Table_1.docx]

**Supplementary TABLE 1** R packages used in this Study.

| Statistical analysis | R package |
| --- | --- |
| mRMR | mRMRe |
| LASSO logistic regression | glmnet |
| RFS-FS | randomForest |
| SVM-RFE | e1071 |
| Logistic regression, nomogram, C-index, calibration plot | rms |
| Collinearity diagnosis | car |
| Hosmer-Lemeshow test | vcdExtra |
| DCA curve | ggDCA |
| NRI | nricens |
| IDI | PredictABEL |
